# The association between the weight-adjusted-waist index and frailty in US older adults: a cross-sectional study of NHANES 2007–2018

**DOI:** 10.3389/fendo.2024.1362194

**Published:** 2024-09-09

**Authors:** Shanshan Jia, Xingwei Huo, Lirong Sun, Yuanyuan Yao, Xiaoping Chen

**Affiliations:** ^1^ Cardiology Department, West China Hospital, Sichuan University, Chengdu, Sichuan, China; ^2^ Affiliated Hospital of Xizang Minzu University, Xianyang, Shaanxi, China

**Keywords:** weight-adjusted-waist index, frailty, obesity, NHANES, cross-sectional study

## Abstract

**Objective:**

This study aimed to evaluate the relationship between the weight-adjusted waist circumference index (WWI) and the frailty in American adults aged over 60 years.

**Methods:**

We utilized data from the National Health and Nutrition Examination Surveys (NHANES) spanning from 2007 to 2018. WWI was calculated using the square root of waist circumference (cm) divided by body weight (kg). The frailty index ≥ 0.25 was employed to assess frailty. Weighted multivariate logistic regression was conducted to explore the association between WWI and frailty. Generalized Additive Modeling (GAM) was used to explore potential non-linear relationships. Receiver operating characteristic curve (ROC) analysis was used to assess the predictive ability of WWI for frailty.

**Results:**

The study encompassed 7765 participants. Higher WWI was significantly associated with higher odds of frailty. In the fully adjusted model, each unit increase of WWI was associated with an 82% increased odds of frailty (OR: 1.82, 95% CI: 1.61 – 2.06; P < 0.001). GAM found significant nonlinear relationships and threshold effects.

**Conclusion:**

The study presented a robust correlation between elevated WWI and increased odds of frailty among American older adults. However, these findings require further validation in large-scale, prospective studies.

## 1 Introduction

Frailty represents a multifaceted, age-associated clinical syndrome characterized by diminished physiological capacity across various organ systems, substantially heightening the likelihood of adverse health outcomes (1, 2). This condition is notably linked with a heightened risk of several adverse events, including delirium, falls, hospitalizations, disabilities, fractures, and increased mortality rates (3, 4). In the context of global aging, the incidence of frailty will further increase (2). Therefore, it is essential to identify frailty-related risk factors and propose targeted intervention and preventive measures.

Obesity, resulting from a complex interplay of metabolic, genetic, behavioral, and environmental factors (5), is projected to affect over half of the adult population in 29 U.S. states by 2030 (6). Previous research has linked obesity with accelerated frailty progression (7). BMI is a widely utilized metric for obesity assessment and classification, but studies have found a U-shaped association between BMI and frailty (7, 8). However, it fails to distinguish fat, lean mass, and fat distribution (9). Therefore, it is necessary to explore novel obesity indicators.

The Weight-Adjusted Waist Circumference Index (WWI), introduced by Park et al. (10), is calculated as Waist Circumference/√weight. This index focuses on central obesity while reducing the correlation with BMI (11). Studies have found that increased WWI is independently associated with an increased risk and strong predictive ability for diseases and mortality (12–14). Therefore, WWI may potentially enhance the precision of obesity categorization and risk stratification, facilitating more targeted therapeutic approaches and monitoring strategies.

Despite its clinical importance, the relationship between WWI and frailty, particularly among older adults, remained underexplored. This study examined this association using data from the 2007 to 2018 National Health and Nutrition Examination Survey (NHANES).

## 2 Materials and methods

### 2.1 Database sources and subjects

NHANES, a nationally representative cross-sectional survey conducted by the National Center for Health Statistics (NCHS), employs a stratified, multistage probability sampling method. It assesses the health and nutritional status of the noninstitutionalized civilian population in the United States. The NCHS Research Ethics Review Board approved the NHANES protocol. The dataset, devoid of personal identifiers, is publicly available.

**Figure 1:**
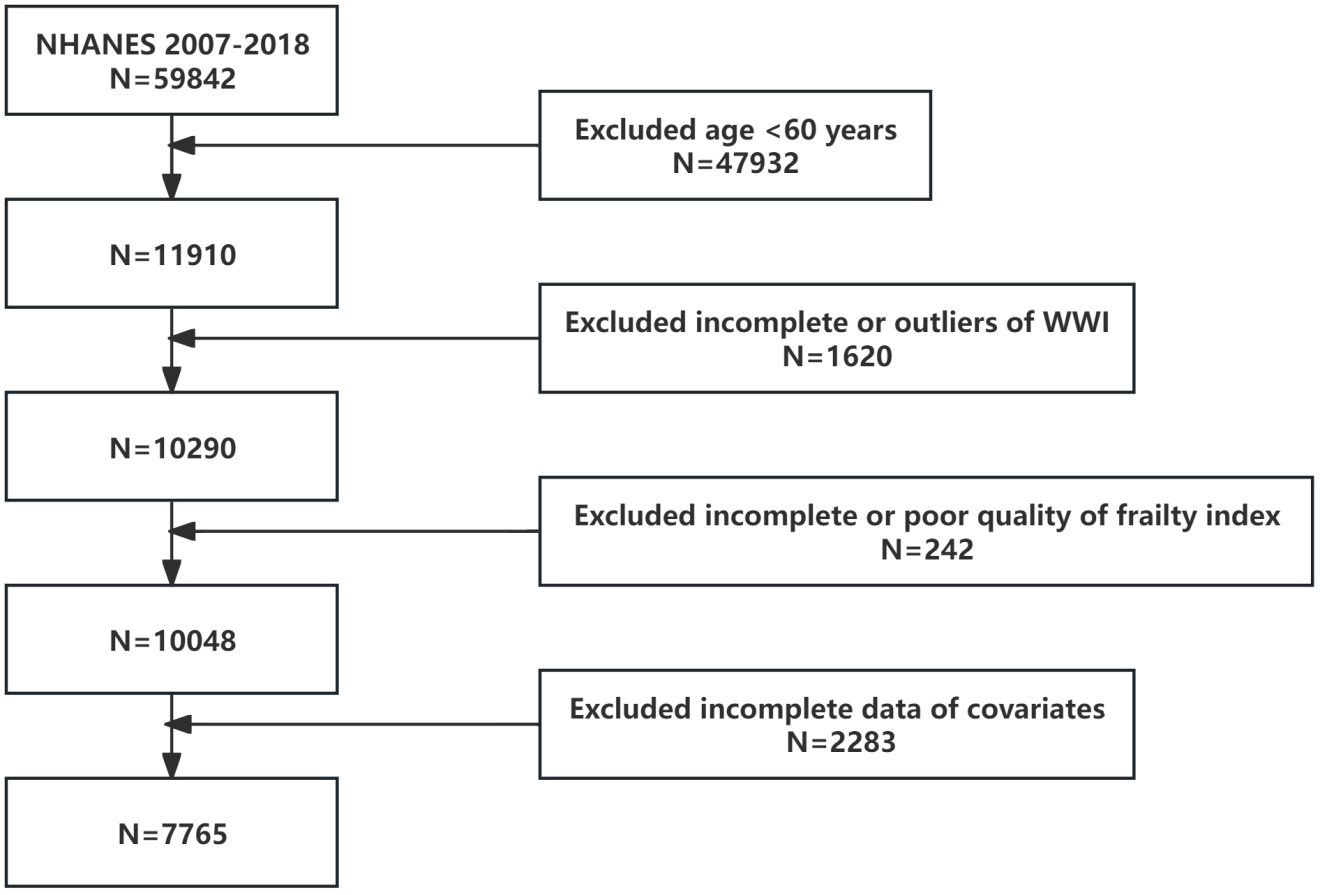
Flowchart of the population selection from NHANES.

For this study, we initially considered data from 59842 participants collected from 2007 to 2018. The final sample comprised 7765 participants (**Figure 1**), excluding those under 60 years (N=47932), those with missing or outliers data of WWI (N=1620), unreliable frailty index assessments (N=242), or missing covariates (N=2283). Comprehensive information is publicly available on the CDC website (https://www.cdc.gov/nchs/nhanes/).

### 2.2 Assessments of frailty

Frailty, the outcome variable, was defined using a frailty index **≥** 0.25 (15). The frailty index was established by Wael Sabbah et al., which incorporates 49 diagnostic criteria following Searle and colleagues’ standard procedures (16, 17). These criteria span a wide range of factors relevant to frailty, including cognitive function, depressive symptoms, daily activity levels, physical performance, presence of chronic diseases, overall health status, healthcare utilization, and results from laboratory tests. A detailed breakdown of these criteria is provided in **Supplementary Table 1**.

Each criterion within the frailty index is scored on a scale from 0 to 1 based on severity. Each participant’s overall frailty index score is then calculated by dividing the sum of all individual scores by the total number of items assessed. Only participants who responded to at least 80% of the index items were included in the analysis to ensure the reliability of the frailty diagnoses.

### 2.3 Assessments of weight-adjusted waist circumference index

WWI, the exposure variable, was calculated as WC in centimeters divided by the square root of body weight in kilograms. To ensure measurement accuracy and consistency, all NHANES staff underwent rigorous training. Additionally, the anthropometric equipment used at each Mobile Examination Center (MEC) was standardized and regularly calibrated.

To maintain the robustness of our conclusions, our analysis excluded participants whose WWI values were greater or less than three standard deviations from the mean. This step was crucial in mitigating the impact of outliers on the study’s findings. WWI was analyzed both as a continuous and categorical variable. Participants were stratified into three groups based on WWI tertiles: tertile 1 (9.36<wwi ≤ 11.22), tertile 2 (11.22<wwi ≤ 11.81), and tertile 3 (11.81<wwi ≤ 13.69).

### 2.4 Covariates

To avoid the influence of confounding factors, we adjusted for known covariates. These confounding factors encompassed age, gender, race, education, marry, poverty income ratio (PIR), smoking, alcohol use, systolic blood pressure (SBP), diastolic blood pressure (DBP), healthy eating index-2015 (HEI-2015) and energy intake.

PIR was categorized as follows: PIR ≤ 1.3 for low income, 1.3 < PIR ≤3.5 for middle income, and PIR > 3.5 for high income. Smoking was defined as a lifetime smoking history of at least 100 cigarettes. Alcohol use was categorized based on current drinking status (18, 19). Non-drinkers were classified as never (less than 12 drinks in lifetime) or former (ceased last year with at least 12 drinks in lifetime). Current drinkers were split into heavy (at least 4 for males, at least 3 drinks/day for females, or binge drinking at least 5 days/month), moderate (at least 3 drinks/day for males, at least 2 drinks/day for females, or binge drinking at least 2 days/month), and mild (current drinking but not meet above standard). Binge drinking is defined as ≥4 drinks for females or ≥5 for males on the same occasion. Blood pressure was averaged from at least three consecutive standard measurements. Dietary data were obtained from the first day 24-hour dietary questionnaire. The HEI -2015 is an indicator of how consistent an individual’s diet quality is with the Dietary Guidelines for Americans (20). HEI-2015 scores range from 0-100, with higher HEI scores reflecting better diet quality and healthier diet. Detailed questionnaire contents and examination methods are available on NHANES’s official website.

### 2.5 Statistical analyses

All statistical analyses accounted for NHANES’ complex sampling design and used appropriate sampling weights. Continuous variables were presented as means ± standard errors (SE), and categorical variables as weighted proportions. Baseline group differences were assessed using weighted t-tests and weighted chi-square tests.

The association between WWI and frailty was examined through weighted multivariate logistic regression with three models: Model 1 was unadjusted; Model 2 adjusted for age, gender, race, and education; Model 3 included adjustments for all covariates.

Generalized Additive Modeling (GAM) evaluated potential nonlinear associations, and two-stage linear regression models explored turning points and threshold effects. Subgroup analyses and interaction tests were also conducted. Receiver Operating Characteristic (ROC) analysis was used to observe the diagnostic ability of WWI for frailty. The DeLong test is used to test the statistical difference of ROC. Sensitivity analysis included two items: 1) further adjusting for BMI and medication conditions such as hypertension, diabetes, cardiovascular disease (CVD), chronic obstructive pulmonary disease (COPD), chronic kidney disease (CKD), and the use of antihypertensive, antidiabetic and antihyperlipidemic drugs; 2) imputing missing covariates using the random forest-based algorithm (21). All analyses were performed using R software (version 4.2.1) and EmpowerStates (version, 4.1), with a two-tailed p-value < 0.05 considered statistically significant.

## 3 Results

### 3.1 Baseline characteristics of participants

**Table 1** presented the baseline characteristics of the study population. The study comprised 7765 participants, with a mean age of 69.19 ± 0.12 years, 45.84% male and 54.16% female. The average WWI was 11.48 ± 0.01. As WWI levels increased, the prevalence of frailty increased significantly. The three groups exhibited markedly distinct profiles among all baseline characteristics.

**Table 1:** The clinical characteristics of participants.

Characteristics	Total	Tertile 1	Tertile 2	Tertile 3	P value
Age (years)	69.19(0.12)	67.73(0.20)	69.31(0.23)	70.75(0.18)	<0.001
**Gender %**					<0.001
Female	54.16(0.02)	49.45(1.17)	49.06(1.05)	64.90(1.30)	
Male	45.84(0.02)	50.55(1.17)	50.94(1.05)	35.10(1.30)	
**Race %**					<0.001
Black	7.87(0.01)	9.95(0.88)	7.42(0.69)	5.96(0.67)	
Mexican	3.82(0.00)	2.14(0.33)	4.32(0.59)	5.24(0.80)	
Other	8.21(0.01)	6.81(0.59)	8.87(0.78)	9.13(0.87)	
White	80.09(0.04)	81.10(1.28)	79.39(1.25)	79.67(1.53)	
**Education level %**					<0.001
Less than high school	16.41(0.01)	10.89(0.87)	17.10(1.17)	22.01(1.26)	
High school or GED	24.59(0.01)	21.51(1.32)	24.16(1.43)	28.56(1.42)	
Above high school	59.00(0.02)	67.60(1.57)	58.74(1.75)	49.42(1.44)	
**Marry %**					<0.001
Married	63.23(0.03)	68.20(1.47)	65.56(1.28)	55.10(1.52)	
Live with other	2.36(0.00)	2.55(0.41)	2.61(0.50)	1.89(0.35)	
Never married	3.70(0.00)	3.94(0.47)	3.12(0.43)	4.05(0.54)	
Other	30.70(0.01)	25.31(1.32)	28.71(1.06)	38.96(1.57)	
**PIR %**					
Low income	16.46(0.01)	11.31(0.75)	16.67(0.94)	22.14(1.23)	
Med income	39.08(0.02)	34.97(1.57)	38.06(1.48)	44.87(1.39)	
High income	44.46(0.02)	53.72(1.86)	45.27(1.64)	32.99(1.76)	
**Alcohol use %**					<0.001
Never	13.14(0.01)	10.71(0.88)	12.42(0.92)	16.67(0.90)	
Former	20.18(0.01)	16.95(0.99)	19.05(1.08)	25.05(1.36)	
Mild	47.82(0.02)	52.99(1.47)	46.96(1.48)	42.80(1.48)	
Moderate	12.31(0.01)	13.01(0.86)	13.88(1.15)	9.85(1.10)	
Heavy	6.56(0.01)	6.34(0.70)	7.69(0.75)	5.63(0.67)	
Smoking %	50.63(0.02)	47.14(1.25)	54.14(1.47)	50.95(1.43)	>0.002
BMI (Kg/m²)	29.29(0.12)	26.58(0.14)	29.33(0.12)	32.34(0.20)	<0.001
WC (cm)	103.06(0.30)	93.62(0.36)	103.93(0.29)	112.95(0.44)	<0.001
SBP (mmHg)	130.86(0.32)	129.24(0.55)	131.30(0.52)	132.26(0.50)	<0.001
DBP (mmHg)	67.94(0.27)	69.93(0.39)	67.90(0.36)	65.69(0.36)	<0.001
HEI-2015	54.09(0.28)	56.03(0.52)	53.73(0.43)	52.26(0.34)	<0.001
Energy (kcal)	1910.25(16.29)	1982.40(24.49)	1931.54(26.23)	1805.34(21.37)	<0.001
**Frailty (%)**					<0.001
No	72.34(0.03)	82.86(1.12)	74.64(1.13)	57.88(1.30)	
Yes	27.66(0.01)	17.14(1.12)	25.36(1.13)	42.12(1.30)	

GED, general educational development; BMI, body mass index; WC, waist circumference; SBP, systolic blood pressure; DBP, diastolic blood pressure; CVD, cardiovascular disease; COPD, chronic obstructive pulmonary disease; CKD, chronic kidney disease. Continuous variables were summarized using means with SE, and categorical variables were presented as proportions with SE.

### 3.2 Multivariate regression analysis

The association between WWI and frailty was assessed using weighted multivariate logistic regression, with results summarized in **Table 2**. The regression analysis revealed a positive correlation in Model 1 (OR: 2.34, 95% CI: 2.08, 2.63) and Model 2 (OR: 2.05, 95% CI: 1.81, 2.33). In Model 3, the fully adjusted model, each unit increase in WWI, and the prevalence of increases by 82% (OR: 1.82, 95% CI: 1.61, 2.06).

**Table 2:** Association between WWI and frailty.

	OR^a^ (95% CI^b^) *P*-value		
	Model 1^c^	Model 2^d^	Model 3^e^
**Continuous**	2.34(2.08,2.63)	2.05(1.81,2.33)	1.82(1.61,2.06)
	<0.001	<0.001	<0.001
**Categories**			
Tertile 1	Reference	Reference	Reference
Tertile 2	1.64(1.36,1.99)	1.48(1.22,1.80)	1.38(1.12,1.70)
	<0.001	<0.001	0.003
Tertile 3	3.52(2.91,4.25)	2.84(2.31,3.48)	2.40(1.95,2.95)
	<0.001	<0.001	<0.001
P for trend	<0.001	<0.001	<0.001

OR^a^, odds ratio;

95% CI^b^, 95% confidence interval;

Model1^c^, adjusted for non covariates;

Model2^d^, adjusted for age, gender, race, and education;

Model3^e^, further adjusted for marry, poverty income ratio, smoking, alcohol use, systolic blood pressure, diastolic blood pressure, healthy eating index-2015 and energy intake.

Additionally, using WWI as a categorical variable (tertiles) in Model 3, the results demonstrated that, compared to the lowest tertile, the highest tertile had a higher prevalence of frailty after adjusting for all covariates (OR=2.40; 95% CI: 1.95, 2.95).

### 3.3 Nonlinear analysis

**Figure 2:**
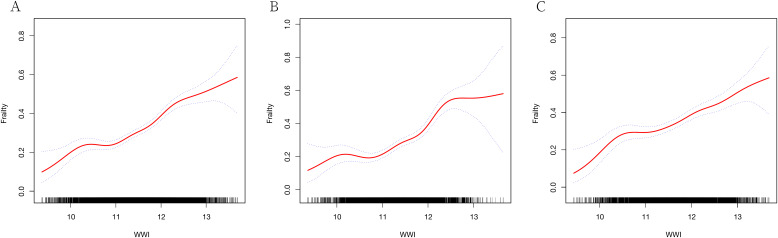
Generalized additive regression. **(A)** GAM for total population; **(B)** GAM for males; **(C)** GAM for females.

GAM indicated a significant nonlinear relationship between WWI and frailty (**Figure 2**). Segmented regression further confirmed the existence of threshold effects (**Table 3**). However, further analysis found that there were differences between genders. The nonlinear effect in males is obvious, and there is a significant threshold effect. In females, the prevalence of frailty increased with WWI, but there was no threshold effect.

**Table 3:** Segmented regression results.

Gender	OR^a^ (95% CI^b^) *P*-value		
	Total	Males	Females
**Segmented Model**			
Turning point (K)	10.98	10.97	10.39
< K OR 1	1.24(0.94, 1.63)	1.08(0.75, 1.55)	5.52(1.35, 22.56)
	0.122	0.691	0.017
> K OR 2	1.90 (1.72, 2.10)	2.50(2.12, 2.93)	1.49 (1.34, 1.67)
	<0.001	<0.001	<0.001
OR 2 - 1	1.54 (1.11, 2.12)	2.32 (1.47, 3.64)	0.27 (0.06, 1.14)
	0.009	<0.001	0.075
Likelihood ratio test	0.010	<0.001	0.055

OR^a^, odds ratio; 95% CI^b^, 95% confidence interval.

### 3.4 Subgroup analysis

Subgroup analyses were conducted to examine the relationship across various demographic and clinical backgrounds, including age, gender, race, BMI, smoking, and alcohol use (**Table 4**).

**Table 4:** Results of subgroup regression.

Subgroup	OR (95% CI)	P	P for interaction
Age			0.025
<75	1.95(1.66,2.30)	<0.001	
≥75	1.57(1.31,1.89)	<0.001	
Gender			0.003
Female	1.62(1.42,1.85)	<0.001	
Male	2.33(1.88,2.89)	<0.001	
Race			0.449
Black	1.68(1.41,1.99)	<0.001	
Mexican	1.63(1.36,1.95)	<0.001	
Other	2.02(1.46,2.79)	<0.001	
White	1.84(1.57,2.15)	<0.001	
BMI			0.015
<25	1.35(1.09,1.67)	0.007	
≥25	1.84(1.55,2.19)	<0.001	
Smoking			0.104
No	1.65(1.41,1.92)	<0.001	
Yes	2.05(1.71,2.46)	<0.001	
Alcohol use			0.017
No	1.40(1.13,1.73)	0.003	
Yes	1.94(1.67,2.25)	<0.001	

Results of the subgroup analysis were adjusted for all covariates except the effect modifier.

The subgroup analysis results confirmed the significant association between WWI and frailty across different demographic backgrounds. Additionally, the interaction tests indicated that the association was more pronounced among individuals under the age of 75, male, with a BMI exceeding 25, and drinkers. However, among different racial groups, this difference was not statistically significant.

### 3.5 Sensitivity analysis

To further verify the robustness of the results, we performed sensitivity analysis: 1) further adjusted for BMI, medication conditions and drug uses; 2) performed imputation for missing covariates. The results of all sensitivity analyses indicated that the association between WWI and frailty is stable. All sensitivity analysis results are in **Supplementary Tables**.

### 3.6 ROC analysis

**Figure 3:**
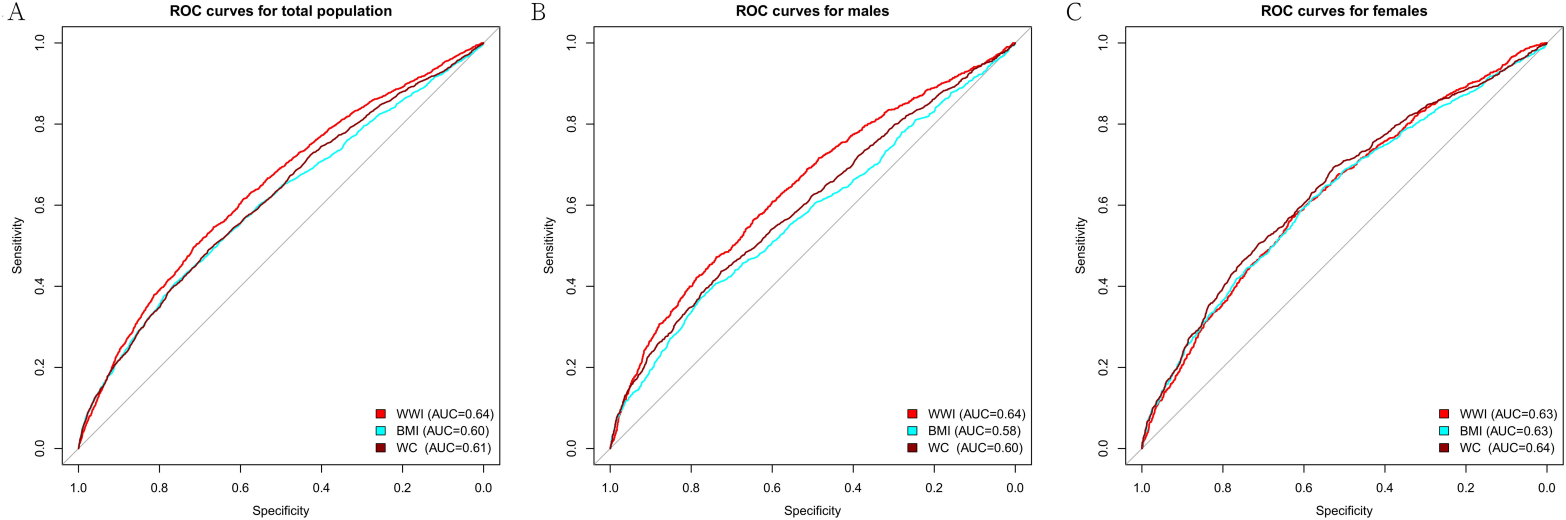
ROC curve of WWI, BMI, and WC. **(A)** ROC for total population; **(B)** ROC for males; **(C)** ROC for females. WWI, weight-adjusted waist index; BMI, body mass index; WC, waist circumference.

We performed ROC analysis to compare the predictive power of WWI with BMI and WC for frailty. It was found that WWI had a stronger predictive ability for frailty than BMI and WC in the total population (**Table 5**). This effect appeared to be more pronounced in males. However, WWI, BMI, and WC did not show statistical differences in their ability to predict frailty among females (**Figure 3**).

**Table 5:** ROC analysis results.

	Variable	AUC (95% CI)	Threshold	Sensitivity	Specificity	Youden Index	P value
Total	WWI	0.64(0.63,0.65)	11.76	0.50	0.71	0.21	–
	BMI	0.60(0.59,0.62)	31.54	0.41	0.76	0.17	<0.001
	WC	0.61(0.60,0.63)	106.25	0.49	0.68	0.17	<0.001
Males	WWI	0.64(0.62,0.66)	11.69	0.47	0.74	0.21	–
	BMI	0.58(0.56,0.60)	31.38	0.37	0.78	0.15	<0.001
	WC	0.60(0.58,0.62)	110.25	0.44	0.73	0.16	<0.001
Females	WWI	0.63(0.61,0.65)	11.76	0.57	0.63	0.20	–
	BMI	0.63(0.61,0.64)	28.99	0.62	0.58	0.20	0.739
	WC	0.64(0.62,0.66)	97.55	0.69	0.53	0.22	0.143

WWI, weight-adjusted waist index; BMI, body mass index; WC, waist circumference.

## 4 Discussion

This investigation represented the first cross-sectional analysis to explore the relationship between WWI and frailty, utilizing data from the NHANES. The study included 7765 participants, revealing a higher prevalence of frailty in individuals with elevated WWI. This association persisted consistently even after adjusting for all covariates. GAM identified the nonlinear association and gender differences. ROC analysis indicated that WWI had superior predictive value for frailty, particularly among males.

The interaction test results showed that the association was more significant among those who were age <75, male, BMI ≥25, and drinkers. Therefore, actively adopting healthy lifestyles to improve WWI for these people may benefit more from reducing the risk of frailty. Despite the interaction, the association was statistically significant in all subgroups. This consistency across diverse subgroups further underscores the robustness and generalizability of our results, indicating that the relationship between WWI and frailty is applicable across a broad spectrum of population segments.

A recent study used BMI and metabolic status to classify obesity in a prospective cohort to explore the role of the metabolic status of obesity in the progression of frailty (22). It demonstrated that the transitioning from a healthy to an unhealthy metabolic status, regardless of obesity, accelerates frailty progression. This underscores the importance of metabolism in frailty and the limitations of BMI in reflecting metabolic status, contributing to the obesity paradox (9, 12).

Recent studies have increasingly highlighted the critical role of abdominal adiposity in the development of frailty. In addition, a cross-sectional study found that abdominal adiposity was most strongly associated with functional scores and had good sensitivity with frailty scores (23). A study involving community-dwelling hypertension elderly people from China has shown that abdominal obesity, as measured by WC, was associated with various forms of frailty, including physical, psychological, and social, whereas total body obesity, as indicated by BMI, was predominantly linked to physical frailty (24). The distinction suggested that the fat accumulation site plays a significant role in frailty manifestations. A study involving 3055 community-dwellers aged 65 and over from the UK Longitudinal Study of Aging further supported these observations (8). It found that individuals with higher WC had a significantly increased risk of frailty across all BMI categories. Notably, this increased risk was also evident among underweight older adults, suggesting that abdominal obesity is a critical marker for frailty screening, independent of overall body weight.

WC is a widely used anthropometric measure correlating positively with visceral fat content (25). Several studies have identified WC as a more effective predictor of frailty than BMI (26). However, it is important to note that WC strongly correlates with BMI, which can complicate the interpretation of obesity-related risks. WWI, a novel obesity indicator, combines the advantages of WC while reducing its association with BMI (14). Moreover, our research indicates that WWI has a stronger predictive ability for frailty than BMI and WC in males.

Several studies provided pivotal insights into the implications of WWI. The Multi-Ethnic Study of Atherosclerosis (MSEA) found that, across all racial groups, an increase in WWI was significantly associated with an increase in abdominal fat and a decrease in muscle mass, with these effects becoming more pronounced with age (27). Further research in Korean community adults supports these findings, demonstrating a close association between elevated WWI and a higher fat mass, lower muscle mass, and reduced bone mass (28). Therefore, WWI is considered a comprehensive body composition index, reflecting fat and muscle mass changes.

In addition, studies have also revealed significant associations between WWI and frailty-related risk factors (12, 29–31). WWI is linked to an increased risk of CVD, including heart failure (30), arterial stiffness (32), left ventricular hypertrophy (33), and hypertension (34). Beyond its cardiovascular implications, WWI has also been associated with neurological issues and cognitive impairment. It is linked to an elevated risk of dementia (35), stroke (14), and depression (36), indicating its broader impact on neurological function and mental health. Moreover, WWI’s impact extends to metabolic-related diseases, encompassing conditions like diabetes (29), hyperuricemia (37), and osteoporosis (38), thereby highlighting its role as a critical marker for both obesity and metabolic disorders. Notably, a ten-year longitudinal study involving older adults in China has further underscored the significance of WWI, confirming its association with all-cause mortality, independent of general cardiovascular risk factors (12). This comprehensive range of associations emphasizes the importance of WWI as a multifaceted and accurate measure of obesity, with far-reaching implications for public health and clinical practice.

However, there is a relative scarcity of studies directly comparing WWI with BMI and WC. WWI exhibited superior predictive ability for all-cause mortality compared to BMI (12). Another survey from NHANES highlighted that WWI is excellent for BMI in predicting heart failure (30). In U.S. adults, there were consistent results for the association of erectile dysfunction (ED), and WWI has greater diagnostic power for ED than BMI and WC (13). Our study aligns with these findings. These results underscore the importance of monitoring and managing obesity, using WWI as a tool, in preventing and treating frailty and related health conditions.

The underlying mechanisms are multifaceted and complex. Central to this relationship is visceral adipose tissue (VAT) accumulation, which plays a pivotal role in metabolic disorders. VAT is not merely fat storage but an active endocrine organ, significantly influencing metabolic processes (39, 40). Research has established that VAT contributes to metabolic dysregulation, primarily through its role in insulin resistance and the secretion of various inflammatory factors, including interleukin-6 (IL-6) and C-reactive protein (CRP). These inflammatory mediators are known to exacerbate systemic inflammation, a critical factor in the development and progression of frailty. Experimental evidence, mainly from animal studies, supports this link. Removing VAT in aged mice has yielded promising results, including reduced cardiac fibrosis and improved myocardial function (41). Moreover, in models of cerebral ischemia, the removal of VAT in aged mice led to a reduction in infarct volume and decreased levels of pro-inflammatory cytokines in the brain, further underscoring the systemic effects of VAT (42). Therefore, controlling VAT accumulation is of paramount importance. Its role as a contributor to metabolic dysregulation and a risk factor for various age-related diseases, including frailty, highlights the need for targeted interventions aimed at managing VAT. This approach could significantly improve overall health outcomes, particularly in the context of aging populations.

Our study also observed gender differences in WWI’s nonlinear analysis and predictive power for frailty. These differences may be attributed to distinct fat accumulation patterns and metabolic differences between genders (43). In males, fat predominantly accumulates in the abdominal area, primarily as VAT, whereas in females, fat tends to deposit in the buttocks, thighs, and other regions, mainly as subcutaneous fat (44). Some studies have found that the accumulation of VAT mass is not linear (45, 46). When total fat reaches a certain threshold, visceral fat begins to accumulate rapidly. The threshold was 23.4% in males and 38.3% in females. These findings suggest a more complex relationship between total body fat and the accumulation of VAT, varying significantly between genders. In addition, metabolic differences cannot be ignored. Estrogen in females promotes fat distribution into peripheral subcutaneous adipose tissue (47). In contrast, testosterone in males tends to direct fat accumulation to the abdominal and visceral regions (43). In light of these findings and our research results, we hypothesize that WWI may offer more significant benefits for obesity management in males. This hypothesis is based on the observation that WWI appears more closely aligned with the fat distribution patterns and metabolic differences prevalent in males. However, the gender difference must be explored in larger prospective cohort studies.

Fortunately, recent studies have shown that frailty is not a static condition but a dynamic process that can be reversed. Our findings indicated that WWI has a stronger predictive power for frailty than BMI and WC without exhibiting a U-shaped association. The identification of the threshold effect in WWI suggests its utility in frailty screening and prevention within reasonable ranges of obesity. Consequently, interventions focused on WWI management may significantly enhance individual health status and considerably impact public health. Future longitudinal studies are warranted to investigate the effects of WWI interventions on the onset and progression of frailty.

While this investigation was comprehensive, encompassing a diverse racial demographic and a large sample size, and focused on linear and non-linear associations, several limitations must be acknowledged. The sample was primarily drawn from U.S. older adults, which may limit the generalizability of the findings to different demographic settings. In addition, it is worth noting that the NHANES study did not include hospitalized and homeless individuals who may be at higher risk of frailty. Additionally, the cross-sectional study cannot establish causal relationships. Diet data may suffer from recall bias. Not all potential confounders were taken into account. ROC analysis was performed to compare the diagnostic value of different indicators rather than to build a predictive model. But we will build an observation model in future research. All in all, large prospective cohort studies are necessary to elucidate the relationship between WWI and frailty further.

## 5 Conclusion

This study demonstrated a significant positive correlation between WWI and frailty. These findings underscored the importance of WWI as a potential tool in frailty assessment and management. However, further research is essential to verify it.

## Data Availability

Publicly available datasets were analyzed in this study. This data can be found here: www.cdc.gov/nchs/nhanes.
